# Cardiac *FGF23* expression correlates with left ventricular hypertrophy in patients with chronic kidney disease

**DOI:** 10.1186/2194-7791-1-S1-A25

**Published:** 2014-09-11

**Authors:** Maren Leifheit-Nestler, Robert Große Siemer, Kathrin Flasbart, Dagmar-Christiane Fischer, Michael Klintschar, Jan U Becker, Christoph Aufricht, Tomas Seeman, Dieter Haffner

**Affiliations:** Department of Paediatric Kidney, Liver and Metabolic Diseases, Hannover Medical School, Hannover, Germany; Department of Paediatrics, University Hospital Rostock, Rostock, Germany; Institute for Forensic Medicine, Hannover Medical School, Hannover, Germany; Institute of Pathology, Hannover Medical School, Hannover, Germany; Division of Paediatric Nephrology, University Children’s Hospital Vienna, Vienna, Austria; Division of Paediatric Nephrology, University Children’s Hospital Motol, Prague, Czech Republic

Left ventricular hypertrophy (LVH) is the most common cardiac abnormality in children with CKD. Experimental and clinical studies demonstrated an association between elevated serum levels of fibroblast growth factor 23 (FGF23) and LVH in CKD. The aim of our study was to investigate i) the endogenous expression of *FGF23* in heart tissue of paediatric CKD patients, ii) to establish the relationship between cardiac *FGF23* expression and typical molecular mechanisms for LVH, and iii) to evaluate whether cardiac *FGF23* expression is associated with LVH and other clinical parameters.

We conducted a retrospective case-control-study in 25 deceased paediatric patients (age 11±8y) with CKD stage 5 and 25 age and sex-matched healthy controls. Myocardial autopsy samples of the left ventricle (LV) were evaluated by immunohistochemistry and qPCR with respect to endogenous *FGF23* expression, calcineurin-NFAT signalling, genes regulating cardiac remodelling, and brain natriuretic peptide (*BNP*) as a marker of LVH.

Cardiac *FGF23* expression increased in CKD patients compared to controls (*p*<0.01), and correlated with LVH demonstrated by enhanced *BNP* expression as well as increased cardiomyocyte cross-sectional area (each *p*<0.01). The calcineurin expression significantly enhanced in cardiac tissue of CKD patients presenting with LVH (*p*<0.05), and cardiac remodelling was confirmed by the enhanced expression of skeletal α-actin and the significant reduction of cardiac α-actin compared to controls (*p*<0.05). In the patient cohort, cardiac *FGF23* expression negatively correlated with the eGFR (r=-0.526), and positively correlated with time-averaged serum phosphate levels (r=0.503, each *p*<0.05). Likewise, cardiac *FGF23* expression was not increased in transplanted patients compared to controls.

For the first time, endogenous *FGF23* expression was detected in myocardial tissue. Cardiac *FGF23* expression as well as the FGF23 signalling pathway mediating cardiac hypertrophy and hypertrophic gene programs were induced in LVH positive CKD patients. We conclude that, in extension to circulating FGF23 levels, both, endogenous and circulating FGF23 contribute to LVH in CKD.

